# Wave-driven butterfly distribution of Van Allen belt relativistic electrons

**DOI:** 10.1038/ncomms9590

**Published:** 2015-10-05

**Authors:** Fuliang Xiao, Chang Yang, Zhenpeng Su, Qinghua Zhou, Zhaoguo He, Yihua He, D. N. Baker, H. E. Spence, H. O. Funsten, J. B. Blake

**Affiliations:** 1 School of Physics and Electronic Sciences, Changsha University of Science and Technology, 2nd Section, South Wanjiali Road #960, Yuhua District, Changsha, Hunan 410004, China; 2 Chinese Academy of Sciences Key Laboratory for Basic Plasma Physics, University of Science and Technology of China, Hefei, Anhui 230026, China; 3 Center for Space Science and Applied Research, Chinese Academy of Sciences, Beijing 100190, China; 4 Laboratory for Atmospheric and Space Physics, University of Colorado, Boulder, Colorado 80303, USA; 5 Institute for the Study of Earth, Oceans, and Space, University of New Hampshire, Durham, New Hampshire 03824-3525, USA; 6 ISR Division, Los Alamos National Laboratory, Los Alamos, New Mexico 87545, USA; 7 The Aerospace Corporation, Los Angeles, California 90245-4609, USA

## Abstract

Van Allen radiation belts consist of relativistic electrons trapped by Earth's magnetic field. Trapped electrons often drift azimuthally around Earth and display a butterfly pitch angle distribution of a minimum at 90° further out than geostationary orbit. This is usually attributed to drift shell splitting resulting from day–night asymmetry in Earth's magnetic field. However, direct observation of a butterfly distribution well inside of geostationary orbit and the origin of this phenomenon have not been provided so far. Here we report high-resolution observation that a unusual butterfly pitch angle distribution of relativistic electrons occurred within 5 Earth radii during the 28 June 2013 geomagnetic storm. Simulation results show that combined acceleration by chorus and magnetosonic waves can successfully explain the electron flux evolution both in the energy and butterfly pitch angle distribution. The current provides a great support for the mechanism of wave-driven butterfly distribution of relativistic electrons.

## Abstract (web summary)

Relativistic electrons trapped in the Van Allen radiation belts sometimes exhibit a minimum of their pitch angle distribution at 90°. Here, the authors explain the origin of this phenomenon in terms of chorus and magnetosonic waves through simulations and observations of a geomagnetic storm data.

## Graphical abstract

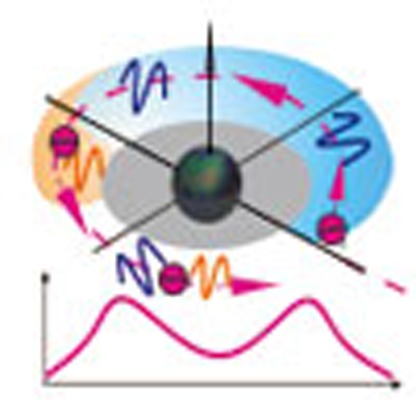

The Earth's outer Van Allen radiation belt is composed of trapped electrons with relativistic energy (*E*_*k*_≥1 MeV). Fluxes and pitch angle distributions of relativistic electrons often exhibit dramatic and highly dynamic changes during geomagnetic storms or substorms, which are associated with different physical mechanisms. Those mechanisms include transport, energization and loss processes. Wave–particle interaction plays an important role in energy exchange between various modes of plasma waves and Van Allen radiation belt relativistic electrons. Two types of plasma waves, chorus and magnetosonic (MS) wave (also known as ‘equatorial noise'), can efficiently accelerate electrons up to relativistic energies. Relativistic electrons can pose serious damage to satellites and astronauts in space, it is therefore important to understand and ultimately predict Van Allen radiation belt electron dynamic variations.

Radiation belt trapped electrons experience azimuthal drift-motion around Earth when they bounce between northern and southern magnetic mirror points. Due to the day–night azimuthal asymmetry in Earth's magnetic field, their drift shells which are traced out by their guiding centres can separate radially for different pitch angles, which is known as drift shell splitting^1^. Significant drift shell splitting occurs only in the outer radiation belt *L*>6 (with *L* being the radial distance in Earth radii *R*_E_), where the asymmetry becomes substantial. Previous theoretical^1^,^2^ and observational^3^,^4^,^5^ works have long confirmed drift shell splitting.

Drift shell splitting separates the high and low pitch angle particles in nightside injections as they move to the dayside magnetosphere. The higher pitch angle particles drift to larger radial distance beyond the magnetopause on the dayside and may consequently be lost from the distribution^6^. Therefore, drift shell splitting can be interpreted in terms of butterfly pitch angle distributions, namely, a sharp dropout in the flux of 90° electrons. Another drift shell splitting is in combination with a negative radial flux gradient as equatorially mirroring particles drift around Earth at locations *L*=6–12 (refs 7, 8). Drift shell splitting will most easily affect trapped particle populations further out than geosynchronous orbit. Occurrence of drift shell splitting inside of geosynchronous orbit would need a very large magnetopause compression during very large geomagnetic storms.

A fundamental problem, both theoretically and observationally, is that there is any butterfly pitch angle distribution of relativistic electrons below *L*=5 associated with a new mechanism instead of drift shell splitting. Previous studies have analysed the characteristics and evolution of pitch angle distributions of the outer radiation belt electrons^9^,^10^. Chorus and MS waves were proposed to produce butterfly distributions by preferentially accelerating off-equator electrons^11^,^12^. Adiabatic processes could potentially yield the formation of butterfly distributions at locations *L*≥6 (refs 13, 14). Direct confirmation of wave-driven butterfly distributions below *L*=5 requires simultaneous high-resolution data but this is generally unavailable before the launch of NASA's Van Allen Radiation Belt Storm Probes in 2012 (ref. 15). Fortunately, the unique events on the 28 June 2013 geomagnetic storm observed from Van Allen Probes provide an excellent opportunity to identify such mechanisms.

Here we report the formation of a unusual butterfly pitch angle distribution of relativistic electrons around *L*=4.8, corresponding to the occurrence of strong chorus and MS waves at the same time. Using two-dimensional simulation, we show that flux enhancements of relativistic electrons are most pronounced between the medium pitch angles 30° and 60° due to the dominant acceleration process for combined chorus and MS waves. Meanwhile, the pitch angle distributions close to 90° are increased due to relatively larger acceleration process for chorus alone. The combined acceleration by chorus and MS waves substantially modifies the whole population of relativistic electrons, producing the butterfly distribution. Our detailed modelling, together with the correlated Van Allen Probes data, presents a further understanding on how chorus and MS waves play different roles in Earth's outer radiation belts.

## Results

### Correlated Van Allen Probe data

**Figure 1:**
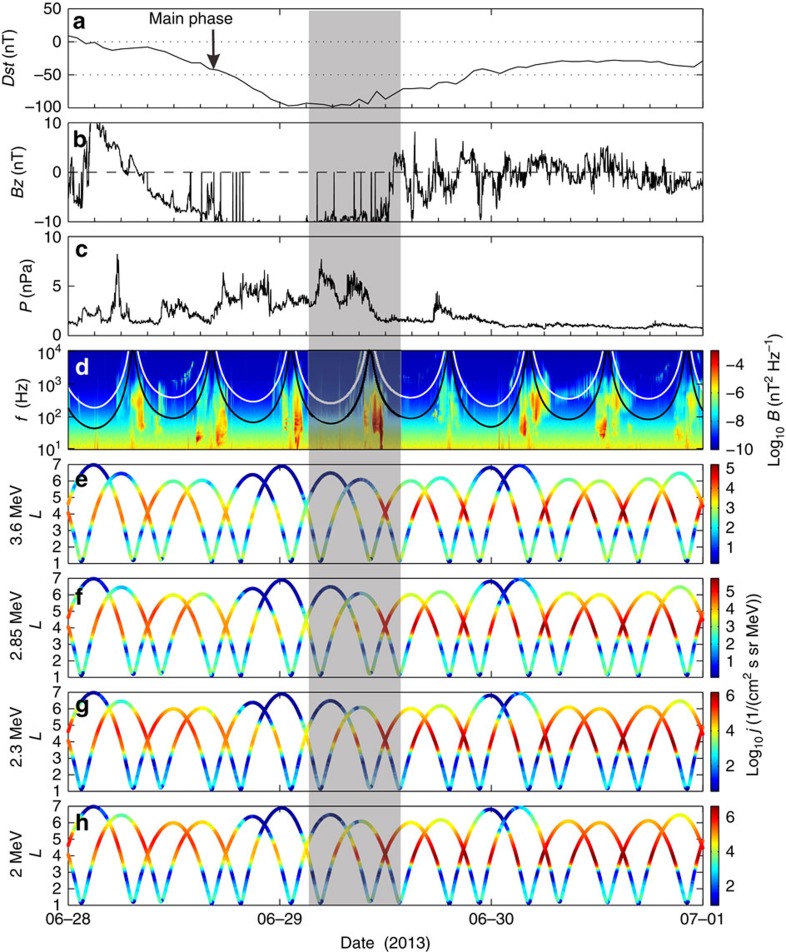
Van Allen Probe data during the 28–30 June 2013 storm. (**a**) The *Dst* index. (**b**) The interplanetary magnetic field *B*_*z*_. (**c**) Solar wind dynamic pressure. (**d**) Magnetic spectral intensity of chorus and MS waves from the EMFISIS instrument on Van Allen Probe A. The black and white lines denote the lower hybrid resonance frequency *f*_lhr_ and 0.1*f*_ce_ (*f*_ce_ being the electron gyrofrequency). (**e**–**h**) Relativistic electron differential fluxes from the REPT instrument onboard the Van Allen Probe A as a function of *L* showing a rapid increase in the radial range 3.5<*L*<5.5 where strong chorus and MS waves occur. The light grey shading area indicates the simulation period.

On 28 June 2013, a moderate storm with Dst≈−100 nT (Fig. 1a) was triggered by an interplanetary coronal mass ejection^16^. This is a relatively minor magnetopause compression, thus drift shell splitting is not expected to occur inside of geosynchronous orbit. A large negative *B*_*z*_ (the *z* component of the interplanetary magnetic field) occurs during the period 1100 hours on June 28 to 1200 UT on June 29 (Fig. 1b), leading to efficient coupling with Earth's magnetosphere and prolonged geomagnetic activity^17^. Distinct whistler-mode chorus and MS emissions (Fig. 1d) were observed by the Electric and Magnetic Field Instrument Suite and Integrated Science (EMFISIS) Waves instrument 18,19 onboard Van Allen Probe A. Chorus and MS waves are right hand polarized electromagnetic waves. Chorus waves are excited by the injection of energetic (tens of keV) plasma sheet electrons into the inner magnetosphere during the period of enhanced plasma convection associated with the intervals of negative *B*_*z*_ (refs 20, 21, 22). MS waves are generated by a ring distribution of low-energy (∼10 keV) protons at frequencies close to the harmonics of the proton gyrofrequency^23^.

The fluxes of relativistic (2–3.6 MeV) electrons (Fig. 1e–h) from the Relativistic Electron-Proton Telescope (REPT) instrument^24^ onboard Van Allen Probes decreased during the main phase of the storm when the magnetopause was compressed down to *L*=7.6, and dramatically increased during the recovery phase after 29 June when the solar wind dynamic pressure decreased and the magnetopause moved beyond *L*=10 (Fig. 1c).

**Figure 2:**
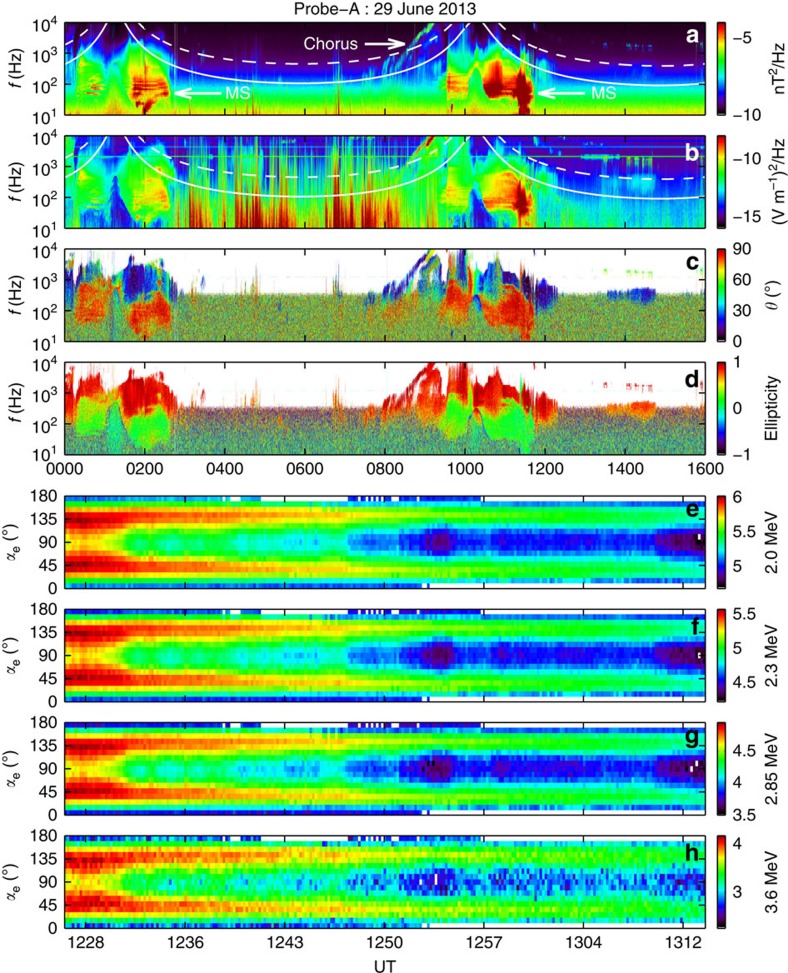
Formation of butterfly distribution of relativistic electrons. Data from the EMFISIS instrument for magnetic and electric spectral intensity (in unit of log_10_) of chorus and MS waves (**a**,**b**), wave normal angle (**c**), the angle between Earth's magnetic field and the normal to the plane of the wave, and wave ellipticity (**d**), the degree of elliptical polarization. Chorus wave occurs above 0.1*f*_ce_ (the white dashed line). MS wave occur as a series of narrow tones, spaced at multiples of the proton gyrofrequency *f*_cp_ up to *f*_lhr_ (the white solid line). (**c**,**d**) The observed MS or chorus wave has a high (*θ*≈90°) or low (*θ*≈0°) normal angle and a high (ellipticity≈0) or low (ellipticity≈1) degree of elliptical polarization. (**e**–**h**) Pitch angle distribution for different indicated energies (2–3.6 MeV) over ∼40 min duration from the REPT instrument. Fluxes (in the same unit as that in Fig. 1) of relativistic electrons have minima around pitch angle 90°, and peaks around the pitch angle range of 30°–60° or 120°–150°.

In Fig. 2, we show data during the period 000 to 1600 UT on 29 June 2013 (corresponding to the shaded area in Fig. 1) from the EMFISIS instrument for magnetic and electric spectral intensity of chorus and MS waves (Fig. 2a,b), wave normal angle *θ* (Fig. 2c) and wave ellipticity (Fig. 2d). The observed chorus wave has a low normal angle (*θ*≈0°) and circular polarization (ellipticity≈0)^25^. Meanwhile, the observed MS wave is highly oblique (*θ*≈90°) and linearly polarized (ellipticity≈0)^26^. This indicates that the MS or chorus wave **k** vector is approximately perpendicular or parallel to the ambient magnetic field direction^12^.

A very interesting feature here is that distinct butterfly distributions of fluxes of relativistic (2–3.6 MeV) electrons from the REPT instrument occurred around *L*=4.8 over the interval 1228-1312 UT (Fig. 2e–h), corresponding to the occurrence of enhanced chorus and MS waves. Electron fluxes have minima around pitch angle 90°, and peaks around the pitch angle range of 30°–60° or 120°–150°. Observations of butterfly distributions at locations *L*<5 are unlikely due to drift shell splitting as Earth's magnetic field is more dipolar and only distorted/stretched under extreme geomagnetic conditions. As this was a relatively minor compression and only a moderate geomagnetic storm, it is unlikely that drift shell splitting is the cause of these observations. Here we perform a two-dimensional simulation, together with the high-resolution observations from the Van Allen Probes, to examine whether chorus and MS waves can be responsible for evolution of both the energy and butterfly pitch angle distribution of the observed relativistic electron flux increase.

### Numerical modelling

To model the temporal evolution of the electron distribution function, we need to solve the Fokker–Planck diffusion equation, which is associated with pitch-angle and momentum diffusion coefficients driven by wave–particle interaction. Then evaluation of diffusion coefficients needs specific information of wave amplitudes and spectral properties. In this event, due to the brief UT intervals of Van Allen Probes, the *in situ* measurements of chorus or MS waves were confined to a limited range of magnetic local time (MLT) and *L*-shells: *L*=4–6 and MLT=22–24 for chorus; *L*=2–3.5 and MLT=10–18 for MS.

Previous works have demonstrated that MS waves can propagate over a broad region of MLT and *L*-shells^23^,^27^,^28^. Here using the previously developed programmes^29^,^30^, we present ray-tracing results of MS waves based on the wave data. MS waves are launched at *L*=5.6 for different parameters and MLT regions at the geomagnetic equator (Supplementary Table 1). The modelled results (Supplementary Fig. 1) confirm that MS waves can propagate either into or out of the plasmasphere through the plasmapause, covering a broad region of *L*=2–5.6, particularly the observed butterfly distribution location *L*=4.8 and MLT=19.

In this event, Van Allen Probes stayed relatively deep inside the plasmasphere in the day–evening sector. Consequently, those MS waves which potentially occurred in the day–evening sector can not be directly observed by Van Allen Probes in their orbits. Since relativistic electrons drift eastward around Earth approximately in a circular orbit, the observed butterfly distribution should come from the whole contribution of resonances with those MS waves in different MLT regions.

We have also checked the Van Allen Probes data on 28 July 2013 and found that MS waves were indeed observed at *L*=4.8 and MLT=19. However, Van Allen Probes only passed the observed location in a brief UT interval every day, they may not observe the MS waves each time. In addition, using the Gaussian fitting method (not shown for brevity), we find that those MS waves display a similar Gaussian distribution to those at *L*=3 and MLT=17.3 on 29 July. It is therefore possibly reasonable to expect that the MS waves potentially existing at *L*=4.8 and MLT=19 on 29 July to follow the similar Gaussian distribution to that at *L*=3 and MLT=17.3.

Meanwhile, for calculating the diffusion coefficients of MS waves, we assume that MS waves propagate very highly oblique (*θ*=86°–89^°^), distributed in a standard Gaussian form (*X*=tan*θ*) with a peak value *X*_m_=tan89°. We noted that a concise modelling method by using maxima wave intensities was adopted in the previous work^26^.

Moreover, chorus occurs over a broad range of MLT from the nightside through dawn to the dayside^31^,^32^, potentially producing efficient scattering precipitation of energetic (tens of keV) electrons into the atmosphere. Considering that the ratio between the precipitated and trapped electron fluxes measured by Polar Orbiting Environmental Satellites (POES) is approximately proportional to the chorus power spectral intensity^33^, previous studies have developed a novel technique to obtain a dynamic global model of chorus wave amplitudes as a function of *L*, MLT and time by converting the measured POES flux ratios at different MLT^34^,^35^. Here we use the same approach (described in Methods), together with the previous parametric study^36^, to obtain the global distribution of chorus wave amplitude as a function of *L*, MLT in this event. Specifically, we remove the proton contamination by the same method in the previous work^37^. The model parameters listed in Table 1 are adopted to calculate diffusion coefficients for chorus waves. However, the current POES model does not incorporate other loss mechanisms and further improvements are needed in the future. Considering that observations of a global distribution of chorus waves are still very limited, this model should move a step forward in constructing an event-dependent global dynamic model of chorus waves.

In general, chorus waves which consist of substructures are coherent at the equator but become less coherent off the equator, resonating with relativistic electrons^38^. The consequences of coherent wave–particle interactions involving relativistic electrons and chorus waves off the equator have been presented in the previous work^39^. Here the quasi-linear theory to treat wave–particle interaction is adopted and the corresponding conditions will be discussed below. We assume chorus waves to obey the same Gaussian distributions in wave frequency and wave normal angle at and off the equator. Considering that relativistic electrons move along the geomagnetic field line and bounce back between mirror points, we consider wave–particle interaction at each location and calculate bounce-averaged diffusion coefficients.

**Figure 3:**
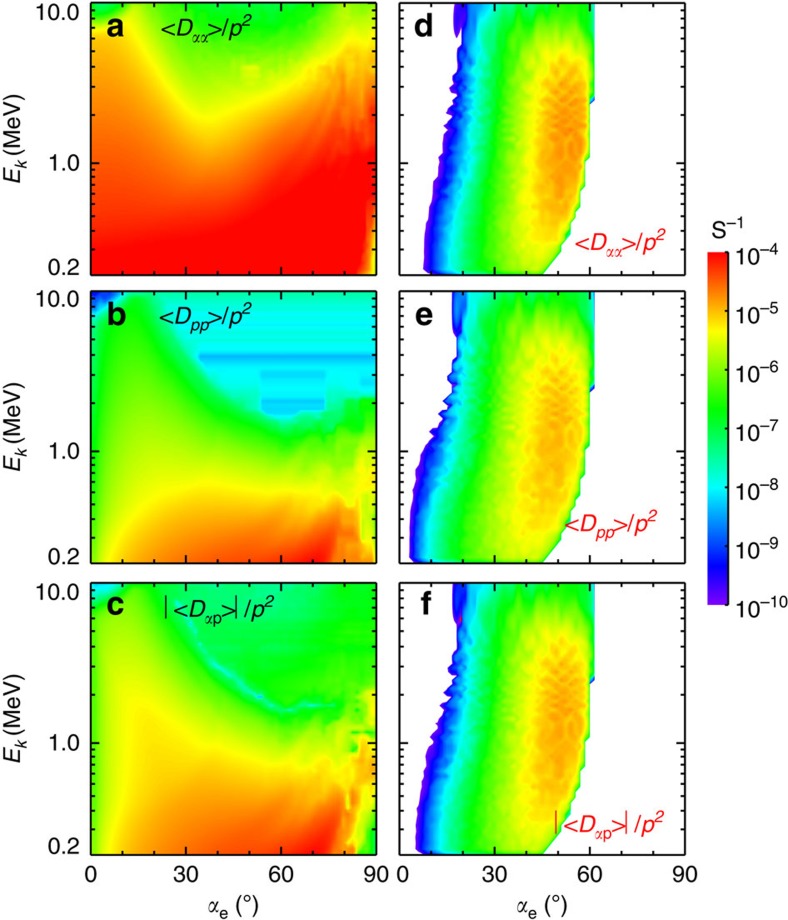
Diffusion rates. Bounce-averaged pitch angle (〈*D*_*αα*_〉, (**a**,**d**)), momentum (〈*D*_*pp*_〉, (**b**,**e**)) and cross (〈*D*_*αp*_〉, (**c**,**f**)) diffusion rates (in unit of s^−1^) for resonant interactions between chorus (left) or MS (right) wave with electrons. Diffusion rates cover an entire region of pitch angles 0°–90° for chorus but a limited region 30°–60° for MS waves. Combination of chorus and MS waves leads to efficient acceleration of electrons between 2 and 3.6 MeV at medium pitch angles 30°–60° and high pitch angles up to 90°, yielding the resultant butterfly distribution on a timescale comparable to 10 h.

The obtained bounce-averaged diffusion coefficients (Fig. 3) cover an entire region of pitch angles 0°–90° for chorus but a limited region 30°–60° for MS waves. This can allow for significant increases in fluxes of relativistic (2–3.6 MeV) electrons at medium pitch angles 30°–60° by MS waves^12^ and at high pitch angles up to 90° by chorus waves^40^, leading to the resultant butterfly distribution on a timescale comparable to 10 h.

**Figure 4:**
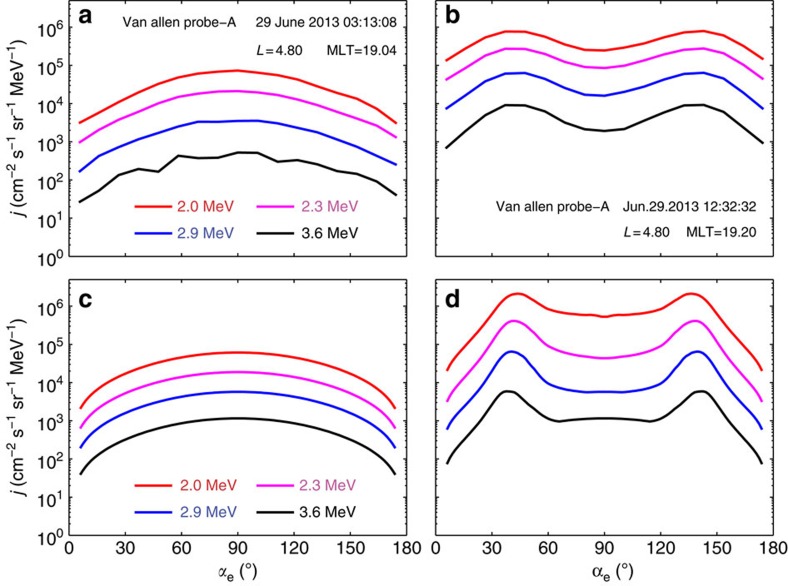
Comparison of Fokker–Planck simulation results with observations. (**a**,**b**) Observed evolution of the relativistic electron fluxes as a function of pitch angle at the same location (shown inside panels) during the simulation. (**c**,**d**) Starting with an initial condition representative of relativistic electrons (**a**), we show the relativistic (2–3.6 MeV) electron fluxes due to acceleration by chorus and MS waves from a numerical solution to the two-dimensional Fokker–Planck diffusion equation. Acceleration is most pronounced within the medium pitch angle region 30°–60° or 120°–150° produced by both chorus and MS waves, and noticeable around the high pitch angles induced by chorus waves. The combined acceleration by chorus and MS waves leads to the butterfly distribution both in the magnitude and the time scale (**d**) comparable to the observation (**b**). The current simulation provides a further support for wave-driven butterfly distribution of relativistic electrons during this storm.

Using aforementioned bounce-averaged diffusion coefficients, we calculate phase space density (PSD) *f*_t_ evolution of electrons in solving a two-dimensional Fokker–Planck diffusion equation^41^. The evolution of differential flux *j* is then simulated in the pitch angle region 0°–90° by the subsequent conversion *j*=*p*^2^*f*_t_ and the results are extended to the range 90°–180° due to the mirror symmetry. We show in Fig. 4a remarkable agreement between the simulated pitch angle distribution (Fig. 4c,d) and the REPT observation (Fig. 4a,b) during the acceleration interval. The most pronounced flux enhancement occurs over the medium pitch angles 30°–60°, where momentum diffusion rates for combined chorus and MS waves dominate (Fig. 3). Furthermore, the pitch angle distributions at higher pitch angles close to 90° are enhanced due to larger momentum diffusion rates of chorus waves. Finally, the combined acceleration by chorus and MS waves significantly alters the whole population of relativistic electrons, yielding the butterfly distribution in about 9 h.

## Discussion

**Figure 5:**
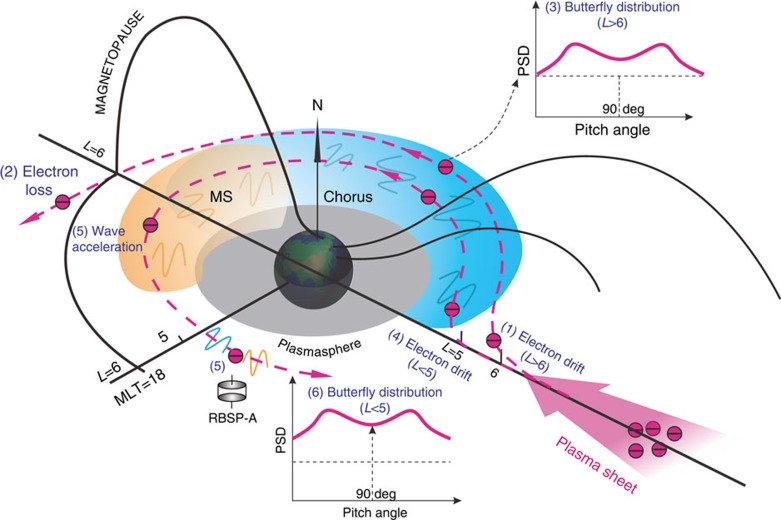
Schematic diagram of formation of butterfly distributions. The long black curves indicate Earth's magnetic field lines. The grey area denote the region of dense cold plasma known as plasmasphere. The blue and yellow areas stand for primary occurrences of chorus and MS waves. The wavy line represents chorus (blue) and MS (yellow) waves. Relativistic electrons originating from the plasma sheet azimuthally drift eastward around Earth. (1) Higher pitch angle electrons at *L*>6 drift to larger radial distance beyond the dayside magnetopause and are consequently lost due to the day–night magnetic field asymmetry (2), yielding the regular butterfly distribution (3). (4) Electrons at *L*<5 drift inside the magnetosphere without loss to the dayside magnetopause because of a very small day–night magnetic field asymmetry, continuously resonating with chorus and MS waves. (5) Wave acceleration can enhance electron PSD primarily within the medium pitch angles, leading to the formation of the unusual butterfly distribution (6).

The present modelling provides a further confirmation of wave-driven butterfly pitch angle distribution of relativistic electrons observed by the REPT instrument at lower locations (*L*<5) of the outer radiation belt. This is in contrast to the formation of butterfly distributions at higher locations (*L*>6) induced by drift shell splitting due to local magnetic field asymmetry. The associated physical processes are schematically presented in Fig. 5. Relativistic electrons at *L*<5 drift azimuthally around Earth inside the magnetosphere without loss to the magnetopause, continuously resonating with chorus and MS waves. The combined acceleration by chorus and MS waves occurs preferably between the medium pitch angles 30° and 60°, leading to the unusual butterfly distribution. The combined acceleration process described here is a universal physical process, which should also be effective in the magnetospheres of Jupiter, Saturn and other magnetized plasma environments in the cosmos.

It should be mentioned that, in a departure from the coherent chorus–electron interaction approach^38^, we use the quasi-linear theory of wave–particle interaction, which has been frequently adopted by radiation belt research community to treat wave–particle interaction^12^,^40^,^42^. Previous work^43^ has shown that energetic electron pitch angle scattering by coherent chorus waves is 3 orders more rapid than by incoherent chorus waves. Coherent chorus waves can produce particle diffusion, phase trapping and/or phase bunching, which is determined primarily by the competing effects of wave amplitude and ambient magnetic field inhomogeneity at resonance. However, application of coherent wave–particle interaction to the strom-time global evolution of energetic particle distributions has not yet been established so far. We therefore expect the adopted quasi-linear theory here to be a basis for comparison with future developments in nonlinear modelling.

The basic conditions for the quasi-linear theory are that each individual particle randomly moves in velocity space, resonates with a succession of uncorrelated and small amplitude waves, and is scattered in a small amount in pitch angle and energy each time. Those conditions are basically satisfied in Earth's radiation belts for naturally generated MS and chorus waves, where the MS wave bandwidth is generally above the proton gyrofrequency up to the lower hybrid frequency and the chorus wave roughly lies in the frequency range 0.1–0.8 *f*_ce_ (*f*_ce_ being the electron gyrofrequency).

**Figure 6:**
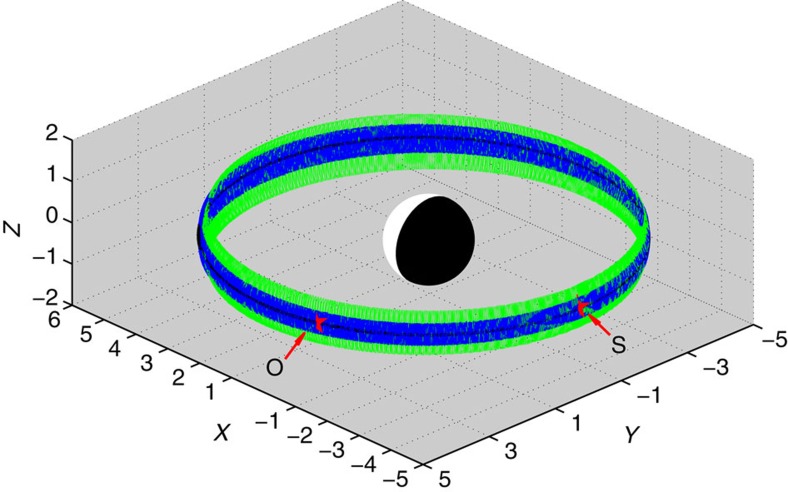
Trajectories of trapped electrons. Test-particle simulations of relativistic (2 MeV) electron trajectories by the TS04 magnetic model for different pitch angles 70° (green), 80° (blue) and 90° (black). The input parameters for TS04 magnetic model are based on the observations. Starting at the location (the symbols *S*): *L*=4.8 and MLT=24, relativistic electrons drift eastward around Earth and approach a farthest location *L*≈5.1 on the dayside. Then they pass the observed location (the symbol *O*) without loss to the magnetopause because the magnetopause locates above *L*=7.6 in the simulation period.

In general, there could be a distorted/stretched geomagnetic field on the nightside during a geomagnetic storm. We have examined the geomagnetic field data from Van Allen Probes and found that the observed data are close to the dipole field model during this storm period. The largest distortion of the dipole field is about 20% in the simulation period. Moreover, we perform a test-particle simulation of trajectories of trapped relativistic (2 MeV) electrons by the TS04 magnetic model^44^ for different high pitch angles. Simulation results (Fig. 6) show that, starting at the location *L*=4.8 and MLT=24, relativistic electrons drift eastward around Earth and approach a farthest location *L*≈5.1 on the dayside. Then they pass the observed location without loss to the magnetopause because the magnetopause locates above *L*=7.6 in the simulation period. Simulations for different energies or pitch angles (not shown for brevity) indeed show similar results. This indicates that drift shell splitting is unlikely to play a role in the observed butterfly electron distribution in this event.

A basic assumption is adopted here that the cold plasma density remains constant and unchanging during the 9-h simulation period. Using the upper hybrid resonance frequency observation from Van Allen Probes, we infer the ambient electron density at locations along Van Allen Probes' orbits and find that the electron density is comparable to the adopted plasma density and does not change much in 9 h. Analogous to the previous work^40^, this assumption is probably reasonable in the absence of simultaneous and continuous local time observations of cold plasma density in the 9-h period.

To check whether the plasmaspheric plume exists, we analyse the potential data from the Time History of Events and Macroscale Interactions During Substorms (THEMIS) spacecraft at *L*=4.8, and find no distinct presence of plasmaspheric plume except a slightly higher electron density in the region around/after MLT=16 in the simulation period. The plasmaspheric plume resulting from time-varying convection generally forms with a more structured distribution of plasma in the dusk region in the storm main phase. Meanwhile, the plume moves eastward (towards later MLTs) in Earth's rotation eastward, and gradually fades/erodes as the strength of convection decreases. As shown in Fig. 1, the simulation period is in the storm recovery phase on 29 June, >24 h after the onset of the storm on 28 June. Hence, the plume probably already faded and moved to the later MLT region. However, further research is still required in the future because there are numerous uncertainties in accurate determination of either the upper hybrid resonance frequency from Van Allen Probes or the THEMIS spacecraft potential.

## Methods

### Ray tracing of MS waves

We perform the ray tracing of MS waves with wave vector **k** and frequency *ω* by the following standard Hamiltonian equations^45^:


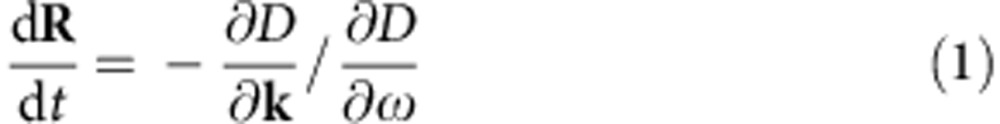



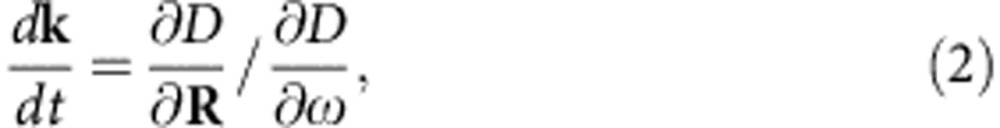


where **R** is the position vector of a point on the ray path, *t* is the group time, *D* represents the standard wave dispersion relation obeying *D*(**R**, *ω*, **k**)=0 at every point along the ray path. The spatial variation in *D* can be written:


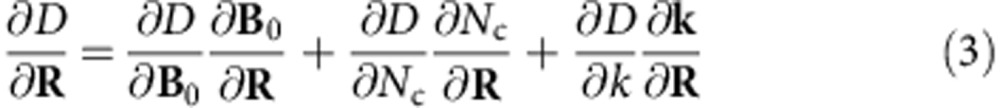


where **B**_0_ is the ambient magnetic field and *N*_c_ is the background plasma density. We adopt a dipole magnetic field model and the MLT-dependent plasmatrough density model^46^. The Earth-centred Cartesian and local Cartesian coordinate systems for the ray-tracing calculation are described in Supplementary Note 1.

### Calculation of the global chorus wave amplitude

Recent works^34^,^35^ have constructed a global chorus wave model based on the data of low-altitude electron population collected by multiple POES satellites. The Medium Energy Proton and Electron Detector (MEPED) onboard POES has two electron solid-state detector (0° and 90°) telescopes to measure electron fluxes in three energy channels (> 30 keV,> 100 keV and>300 keV)^47^. The 90° telescope largely measures the trapped flux over the invariant latitude range between 55° and 70°, and the 0° telescope measures precipitating flux inside the bounce loss cone at *L*>1.4 (ref. 48). Using the electron distribution function near the loss cone^33^, the chorus wave amplitude can be calculated from the ratio between the measured precipitated and trapped electron fluxes (30–100 keV and 100–300 keV). The electron energy spectrum is assumed to follow a kappa-type function^49^,^50^. The basic equation for linking the ratio of electron count rates measured by the 0° and 90° telescopes to chorus wave amplitude is shown in Supplementary Note 2. The ratios of precipitated and trapped electron fluxes measured by multiple POES satellites are used to construct the global chorus wave model over a broad range in *L* and MLT, and the ratios obtained in four distinct MLT sectors are shown in Supplementary Fig. 2.

### Calculation of diffusion coefficients due to chorus and MS waves

We assume that the wave spectral density *B*_f_^2^ follows a typical Gaussian frequency distribution with a center *f*_m_, a half-width *δf*, a band between *f*_1_ and *f*_2_ (ref. 51).


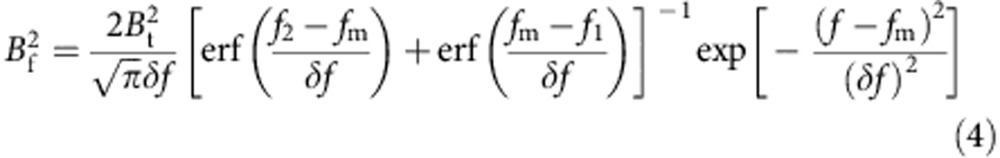


here 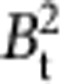 is the wave amplitude in units of Tesla and erf is the error function. To allow the data modelling to be more reliable, we average the observed wave magnetic field intensity over the indicated time period in this event and then apply the corresponding Gaussian fit for MS waves as shown in Supplementary Fig. 3.

We choose the wave normal angle distribution to also satisfy a standard Gaussian form:


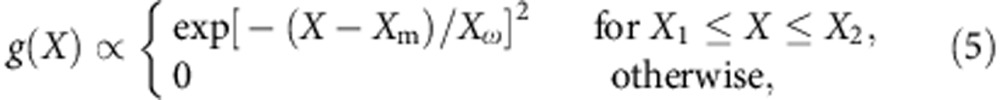


where *X*=tan*θ* (*θ*_1_≤*θ*≤*θ*_2_, *X*_1,2_=tan*θ*_1,2_), with a half-width *X*_*ω*_ and a peak *X*_m_. Based on the observation, we choose *X*_m_=tan 89°, *X*_*ω*_=tan 86°, *X*_1_=*X*_m_−*X*_*ω*_, *X*_2_=*X*_m_+*X*_*ω*_; and the maximum latitude for the presence of MS wave *λ*_m_=10°. Based on the ray-tracing results (Supplementary Fig. 1), we assume the MS wave spectral intensity at *L*=4.8 and MLT=19 hours to follow the similar Gaussian distribution to that as shown in Supplementary Fig. 3, which are then used to calculate the MS-driven bounce-averaged diffusion coefficients. For the chorus waves, we adopt the wave parameters as shown in Table 1 to calculate the chorus-driven bounce-averaged diffusion coefficients at the location *L*=4.8.

We consider contribution from harmonic resonances up to *n*=±5 for both chorus and MS waves. The ambient plasma density is obtained by the MLT-dependent plasmatrough density model^46^ and further assumed to remain latitudinally constant and unchanged during the 9-h simulation period. Note that there is no realistic data available either in 08–12 or in 16–20 MLT. We assume the chorus wave power in 08–12 MLT to follow the latitude-dependent model^36^. The corresponding adopted wave amplitude, wave spectrum and latitudinal occurrence of chorus waves, the ambient electron density and the equatorial ratio of plasma frequency to gyrofrequency (*f*_pe_/*f*_ce_) are shown in Table 1.

### Evaluation of the relativistic electron PSD evolution

The evolution of the electron PSD *f*_t_ is calculated by solving the bounce-averaged pitch angle and momentum diffusion equation


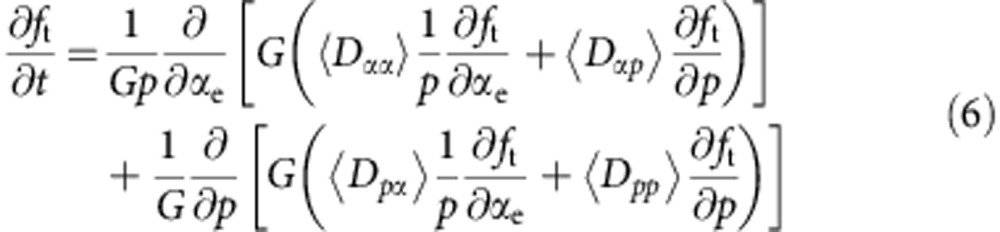


here *p* is the electron momentum, *G*=*p*^2^*T*(*α*_e_)sin*α*_e_cos*α*_e_ with *α*_e_ being the equatorial pitch angle, the normalized bounce time *T*(*α*_e_)≈1.30−0.56sin*α*_e_; 〈*D*_*αα*_〉, 〈*D*_*pp*_〉, and 〈*D*_*αp*_〉=〈*D*_*pα*_〉 denote bounce-averaged diffusion coefficients in pitch angle, momentum and cross pitch angle–momentum. The explicit expressions of those bounce-averaged diffusion coefficients can be found in the previous work.^41^

The initial condition is modelled by a bi-modal kappa-type distribution function of energetic electrons^49^,^50^:


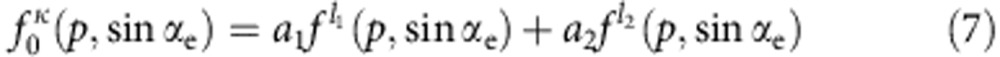


Each component, with variable weighting parameters *a*_1_ and *a*_2_, is expressed as:


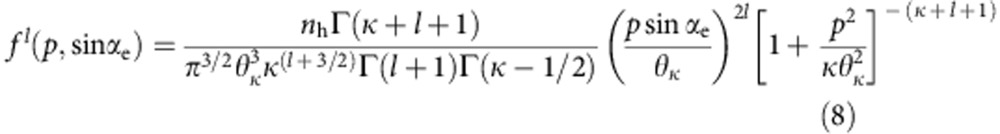


here *n*_*h*_ is the number density of energetic electrons, *l* indicates the loss-cone index, Γ is the gamma function, *κ* and 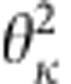 are the spectral index and effective thermal energy scaled by the electron rest mass energy *m*_e_*c*^2^ (∼0.5 MeV).

For the pitch angle boundary condition, *f*_t_=0 at the loss-cone *α*_e_=*α*_*L*_(sin*α*_L_=*L*^−3/2^(4−3/*L*)^−1/4^) to simulate a rapid precipitation of electrons inside the loss cone (Fig. 2e–h), and ∂*f*_*t*_/∂*α*_e_=0 at *α*_e_=90°. For the energy diffusion boundary conditions, 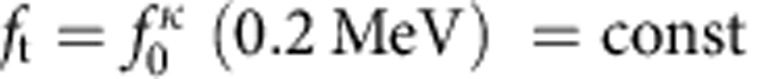 at the lower boundary 0.2 MeV, and 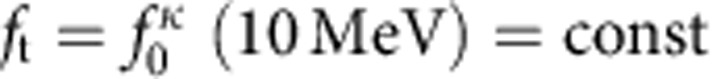 at the upper boundary 10 MeV.

Based on the observation, we choose the following values of parameters: 
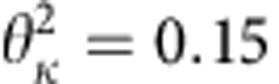
 (∼75 keV), *κ*=4, *n*_*h*_=0.16 cm^−3^; *a*_1_=0.6, *l*_1_=0.6; *a*_2_=0.4, *l*_2_=1.5. We solve the diffusion equation(6) using the recently developed hybrid difference method^41^, which is efficient, stable and easily parallel programmed. The numerical grid is set to be 91 × 101 and uniform in pitch angle and natural logarithm of momentum. The drifting average is taken 25% for MS wave and 1/6 for chorus wave in each aforementioned MLT sector (Table 1).

## Additional information

**How to cite this article:** Xiao, F. *et al*. Wave-driven butterfly distribution of Van Allen belt relativistic electrons. *Nat. Commun.* 6:8590 doi: 10.1038/ncomms9590 (2015).

## Supplementary Material

Supplementary Information Supplementary Figures 1-3, Supplementary Table 1, Supplementary Notes 1-2 and Supplementary References

## Figures and Tables

**Table 1:** Adopted model parameters for chorus^a^.

**MLT**	***f***_**m**_**/*****f***_**ce**_	***δf*****/*****f***_**ce**_	***λ***_**m**_	***N***_**e**_ **(cm**^**−3**^**)**	***f***_**pe**_**/*****f***_**ce**_	***B***_***t***_ **(pT)**
00–04	0.25	0.05	15°	12.2	3.9	63
04–08	0.23	0.05	25°	14.0	4.3	73
08–12	0.21	0.04	45°	20.7	5.2	10^(0.75+0.04*λ*)^
12–16	0.20	0.04	40°	25.6	5.7	36
20–24	0.35	0.05	40°	17.1	4.7	81

MLT, magnetic local time.

No realistic data available either in 08–12 or in 16–20 MLT.

^a^Chorus wave parameters either obtained from direct observations by the Van Allen Probes or inferred from POES data or from the previous parametric result^36^.
