# Supplementary material for: Wave-driven butterfly distribution of Van Allen belt relativistic electrons
Source: Nat Commun. 2015 Oct 5;6:8590. doi: 10.1038/ncomms9590 (PMC4600758; doi:10.1038/ncomms9590)
Supplement: Supplementary Information — Supplementary Figures 1-3, Supplementary Table 1, Supplementary Notes 1-2 and Supplementary References [file ncomms9590-s1.pdf]

Supplementary Figures

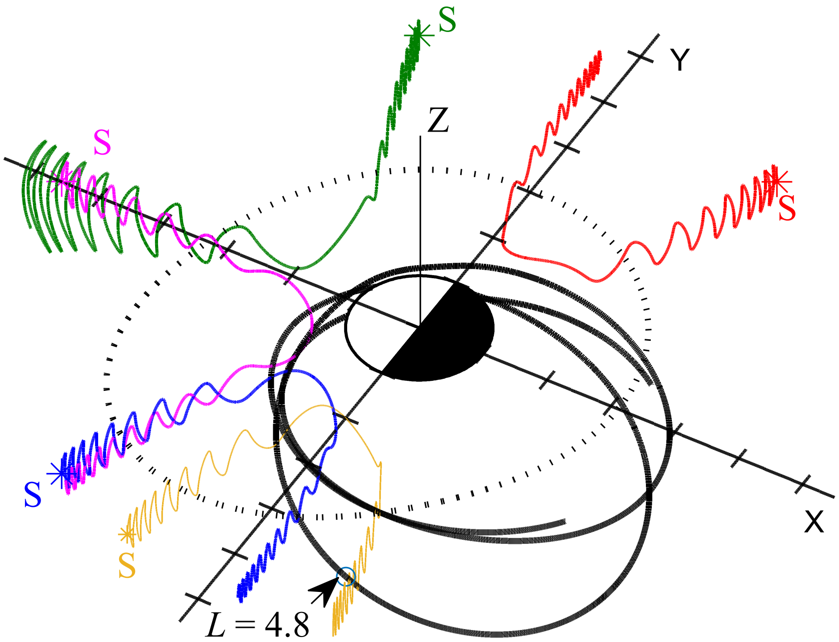

Supplementary Figure 1: Ray-tracing of MS waves. Ray paths representing MS waves are initiated at the geomagnetic equator $L = 5.6$ for different MLT regions (asterisks), with the initial azimuthal angles (η_0) as shown in Supplementary Table 1. The black solid line indicates the trajectory of Van Allan Probe A in this event. The dotted line denotes the plasmapause. MS waves can propagate either into or outside the plasmasphere through the plasmapause, covering a broad region of $L = 2 - 5.6$, particularly the observed butterfly distribution location $L = 4.8$ and MLT=19.

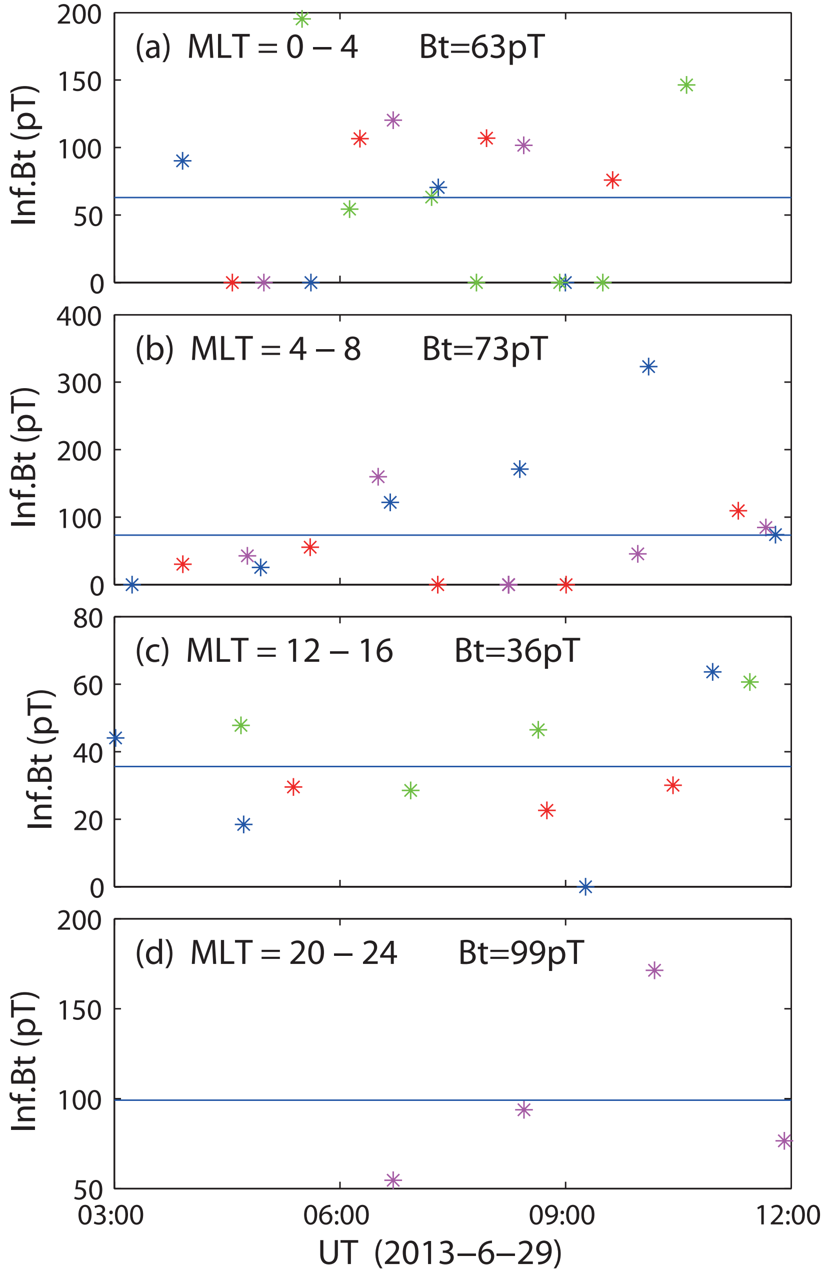

Supplementary Figure 2: Modeling the global distribution of chorus. For the spatial region of $L = 4.8$ in various MLT sectors during $0300 - 1200$ UT on 29 June 2013, the ratios RR, and the inferred chorus wave intensity based on the POES 30-100 keV channel data. Note: the inferred $B_t = 99$ pT in 20-24 MLT is comparable to the observed $B_t = 81$ pT in Table 1.

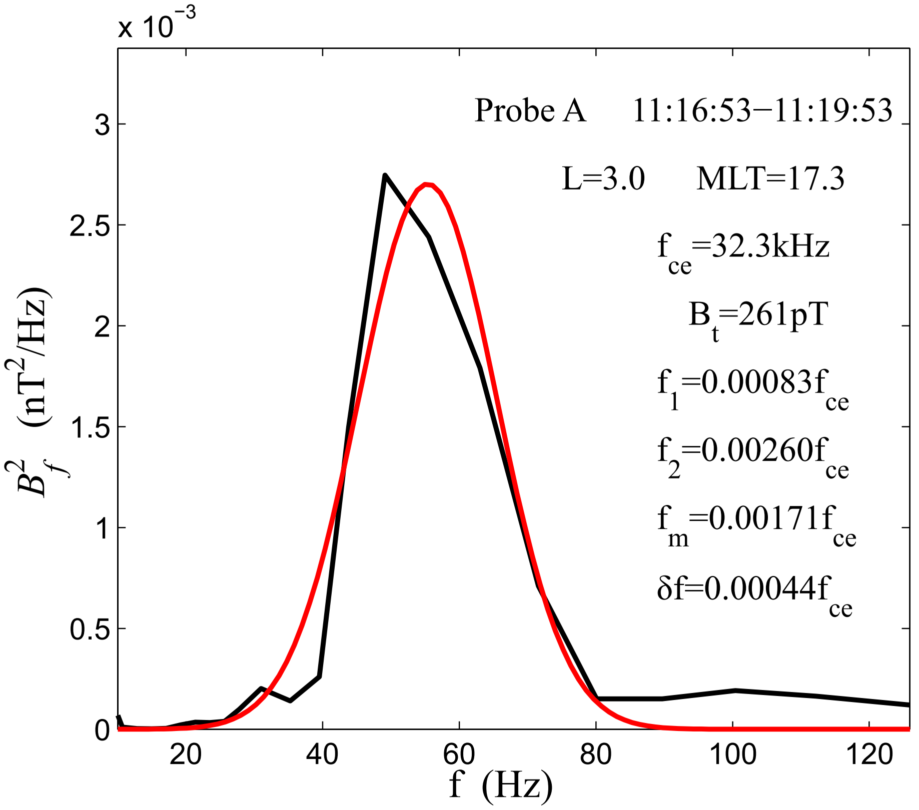

Supplementary Figure 3: Gaussian fitting curves of MS waves. The modeled Gaussian fit (red) to the observed MS wave spectra (black) over a 3-minute period 11:16:53-11:19:53 is shown, together with the fitted wave amplitude B_t, the peak wave frequency f_m, the bandwidth δf, the lower and upper bands f_1 and f_2.

Supplementary Table 1

Supplementary Table 1: Parameters for ray tracing of MS wave

MLT	4	8	12	16	17
η_0	196°	162°	179°	196°	188°

Supplementary Notes

Supplementary Note 1

Here, we use Earth centered Cartesian and a local Cartesian coordinate systems for the ray-tracing calculation. In Earth centered Cartesian coordinate system ($OXYZ$), the Z axis points north along the geomagnetic axis; and the X and Y axes stay in the geomagnetic axis equatorial plane. In local Cartesian system ($pxyz$), the z axis points along the direction of the ambient magnetic field, the x axis is orthogonal to the z axis and stays in the meridian plane pointing away from the Earth at the equator, and the y axis completes the right-handed set. The wave vector $\mathbf{k}$ makes an angle θ with the z axis and the projection of $\mathbf{k}$ onto the xy plane makes an angle η with the x axis, viz., $\mathbf{k} = k\cos\theta\hat{\mathbf{z}} + k\sin\theta\cos\eta\hat{\mathbf{x}} + k\sin\theta\sin\eta\hat{\mathbf{y}}$. $\eta = 0°$, $90°$, $180°$ and $270°$ correspond to the perpendicular component $\mathbf{k}_\perp$ pointing away from Earth, toward later MLT (eastward), toward Earth, and toward earlier MLT (westward), respectively.

Supplementary Note 2

The basic equation for linking the ratio (R) of electron count rates (C.R.) measured by the 0° and 90° telescopes to chorus wave amplitude can be written

$$R = \frac{C.R.|_{0°}}{C.R.|_{90°}} = \frac{\int_{E_1}^{E_2}\int_0^{2\pi}\int_0^{\beta} J_{in}(\alpha, E) A \sin\eta d\eta d\psi dE}{\int_{E_1}^{E_2}\int_0^{2\pi}\int_0^{\beta} J_{out}(\alpha, E) A \sin\eta d\eta d\psi dE} \tag{1}$$

$$= \frac{\int_{E_1}^{E_2}\int_0^{2\pi}\int_0^{\beta} \frac{J(E)}{D_{\alpha\alpha}|_{\alpha_0}\cos\alpha_{in}} \frac{I_0(\frac{\alpha_{in}}{\alpha_0}z_0)}{z_0 I_1(z_0)} \sin\eta d\eta d\psi dE}{\int_{E_1}^{E_2}\int_0^{2\pi}\int_0^{\beta} \frac{J(E)}{D_{\alpha\alpha}|_{\alpha_0}\cos\alpha_{out}} \left[\frac{I_0(z_0)}{z_0 I_1(z_0)} + \ln\frac{\sin\alpha_{out}}{\sin\alpha_0}\right] \sin\eta d\eta d\psi dE} \tag{2}$$

where β and A are the half-angle of the detector acceptance and the sensor area, E_1 and E_2 are lower and upper electron energy (E) for integration, α is the local electron pitch angle given by the cosine law for spherical triangles $\cos\alpha = \cos\theta\cos\eta + \sin\theta\sin\eta\cos\psi$, α_{in} and α_{out} are the equatorial pitch angles corresponding to the local pitch angle α for the 0° and 90° telescopes, J_{in} and J_{out} are electron fluxes measured by the 0° and 90° telescopes, $D_{\alpha\alpha}|_{\alpha_0}$ is the bounce-averaged electron pitch angle diffusion coefficient at the equatorial loss cone α_0 that is mainly controlled by the amplitude of chorus waves, and I_0 and I_1 are modified Bessel functions. $z_0 = \frac{\alpha_0}{\tau_b D_{\alpha\alpha}|_{\alpha_0}\cos\alpha_0}$, where τ_b is a quarter of the electron bounce period. The electron energy spectrum ($J(E)$) is assumed to follow a kappa-type function[1,2] with $\kappa = 5$ and $\theta_\kappa^2 = 0.05$, where κ and θ_κ^2 are the spectral index and effective thermal energy scaled by the electron rest mass energy $m_e c^2$ ($\sim$ 0.5 MeV). More details for inferring the chorus wave amplitudes are shown in the previous work.[3]

Supplementary References

1. Xiao, F., Shen, C., Wang, Y., Zheng, H. & Wang, S. Energetic electron distributions fitted with a relativistic kappa-type function at geosynchronous orbit. *J. Geophys. Res.* 113, A05203 (2008).

2. Vasyliunas, V. M. A survey of low-energy electrons in the evening sector of the magnetosphere with ogo 1 and ogo 3, *J. Geophys. Res.*, 73(9), 2839-2884 (1968).

3. Li, W. et al. Constructing the global distribution of chorus wave intensity using measurements of electrons by the poes satellites and waves by the van allen probes, *Geophys. Res. Lett.*, 40, 4526-4532 (2013).
